# Minimally Invasive Surgery for Inflammatory Bowel Disease: A Systematic Review and Meta-Analysis of Robotic Versus Laparoscopic Surgical Techniques

**DOI:** 10.1093/ecco-jcc/jjae037

**Published:** 2024-03-11

**Authors:** Shafquat Zaman, Ali Yasen Y Mohamedahmed, Widad Abdelrahman, Hashim E Abdalla, Ali Ahmed Wuheb, Mohamed Talaat Issa, Nameer Faiz, Nuha A Yassin

**Affiliations:** Department of General Surgery, Sandwell and West Birmingham Hospitals NHS Trust, Birmingham, UK; College of Medical and Dental Sciences, University of Birmingham, Edgbaston, Birmingham, UK; Department of Colorectal and General Surgery, Royal Wolverhampton NHS Trust, Wolverhampton, UK; Department of Colorectal and General Surgery, Royal Wolverhampton NHS Trust, Wolverhampton, UK; Department of Colorectal and General Surgery, Royal Wolverhampton NHS Trust, Wolverhampton, UK; Department of Colorectal and General Surgery, Royal Wolverhampton NHS Trust, Wolverhampton, UK; Department of General Surgery, Russells Hall Hospital, Dudley Group NHS Trust, Dudley, UK; Department of General Surgery, Russells Hall Hospital, Dudley Group NHS Trust, Dudley, UK; College of Medical and Dental Sciences, University of Birmingham, Edgbaston, Birmingham, UK; Department of Colorectal and General Surgery, University Hospitals Birmingham NHS Trust, Birmingham, UK

**Keywords:** Minimally invasive surgery, robotic surgery, inflammatory bowel disease

## Abstract

**Background:**

We aimed to evaluate outcomes of robotic versus conventional laparoscopic colorectal resections in patients with inflammatory bowel disease [IBD].

**Methods:**

Comparative studies of robotic versus laparoscopic colorectal resections in patients with IBD were included. The primary outcome was total post-operative complication rate. Secondary outcomes included operative time, conversion to open surgery, anastomotic leaks, intra-abdominal abscess formation, ileus occurrence, surgical site infection, re-operation, re-admission rate, length of hospital stay, and 30-day mortality. Combined overall effect sizes were calculated using a random-effects model and the Newcastle–Ottawa Scale was used to assess risk of bias.

**Results:**

Eleven non-randomized studies [*n* = 5566 patients] divided between those undergoing robotic [*n* = 365] and conventional laparoscopic [*n* = 5201] surgery were included. Robotic platforms were associated with a significantly lower overall post-operative complication rate compared with laparoscopic surgery [*p* = 0.03]. Laparoscopic surgery was associated with a significantly shorter operative time [*p* = 0.00001]. No difference was found in conversion rates to open surgery [*p* = 0.15], anastomotic leaks [*p* = 0.84], abscess formation [*p* = 0.21], paralytic ileus [*p* = 0.06], surgical site infections [*p* = 0.78], re-operation [*p* = 0.26], re-admission rate [*p* = 0.48], and 30-day mortality [*p* = 1.00] between the groups. Length of hospital stay was shorter following a robotic sub-total colectomy compared with conventional laparoscopy [*p* = 0.03].

**Conclusion:**

Outcomes in the surgical management of IBD are comparable between traditional laparoscopic techniques and robotic-assisted minimally invasive surgery, demonstrating the safety and feasibility of robotic platforms. Larger studies investigating the use of robotic technology in Crohn’s disease and ulcerative colitis separately may be of benefit with a specific focus on important IBD-related metrics.

## 1. Introduction

The exact aetiology of inflammatory bowel disease [IBD] remains unknown, although in the genetically susceptible individual, an interaction between gut microbiota and the immune system is thought to be important.^1,2^ Refractory IBD is defined as disease not responding to or losing response to immunosuppressants and biologics. This poses a significant challenge to the clinician and a burden to the patient.^3^ Many patients with IBD require surgery with an estimated 10-year risk of up to 10% in ulcerative colitis [UC] and up to 30% in Crohn’s disease [CD].^4^

In patients with UC, the procedure of choice to remove disease burden and offer cure is a staged pan-proctocolectomy with either end ileostomy or ileal pouch-anal anastomosis [IPAA].^5^ The commonest type of surgery in patients with CD is ileo-caecal resection,^6^ although depending on the disease location and mapping, some patients may require surgery to the small bowel or colonic segmental colectomies as well as surgery for the perineum.

Whether it is a limited ileo-caecal resection or a full procto-colectomy, the range of these surgical procedures can be performed either through open surgery or minimally invasive techniques. The advantages of laparoscopic surgery over traditional open operations are well recognized with reduced pain and analgesic requirement leading to earlier mobilization and shorter length of hospital stay. Longer term benefits include better cosmesis, fewer adhesions, and reduced risk of incisional hernia occurrence.^7^

Minimally invasive techniques [laparoscopic surgery] have been applied in the treatment of IBD for the last two decades and are now well established.^8^ The use of laparoscopic surgery has also been shown to be feasible and safe for recurrent CD in specialist centres.^9^ However, the technical challenges of IBD surgery with oedematous tissue planes and shortened mesentery have highlighted the need to use advanced technology to help improve patient outcomes. Robotic surgical platforms have been widely used in colorectal surgery for malignant conditions such as rectal cancer surgery. The benefits seen by utilizing robotic platforms has demonstrated the safe application of this technology in performing complex surgical procedures safely in a minimally invasively manner.^10–12^

At present data on robotic surgery applied to IBD are scarce and of low quality.^13^ However, the improved ergonomics, three-dimensional view, stable camera platform, and dexterity offered by robotic platforms may facilitate its uptake and implementation in IBD surgery.^14^

A previous review of case-matched observational studies comparing robotic with laparoscopic colorectal resections for IBD reported similar outcomes between the two techniques.^15^ A more recent review of non-randomized studies [albeit with limited data] also found that robotic surgery was technically feasible, was safe, and had at least comparable outcomes when compared with laparoscopic IPAA.^16^

We performed a systematic review and meta-analysis of the available literature comparing laparoscopic with robotic colorectal resections in IBD.

## 2. Methods

The eligibility criteria, methodology, and investigated outcome parameters of this study were highlighted in a review protocol which was registered at the International Prospective Register of Systematic Reviews [registration number: CRD42023433049 available at: https://www.crd.york.ac.uk/prospero].

Our review was designed and conducted as per the recommendations of the Cochrane Handbook for Systematic Reviews of Interventions and Preferred Reporting Items for Systematic reviews and Meta-Analyses [PRISMA] guidelines.^17,18^

Papers included in this analysis were based on the following PICO[S] (Population, Intervention, Comparator, Outcomes, [Study design]) format:

- *Population:* patients with an established diagnosis of IBD [CD, UC, indeterminate colitis].
- *Intervention:* any surgical procedure performed for IBD [ileo-colic resection, sub-total colectomy, proctectomy, and IPAA] using a robotic platform.
- *Comparator:* any surgical procedure performed for IBD [ileo-colic resection, sub-total colectomy, proctectomy, and IPAA] using conventional laparoscopy.
- *Outcomes:* post-operative overall complications were our primary outcome. Analysed secondary outcomes included total operative time [minutes], conversion to open surgery, anastomotic leak rate, intra-abdominal abscess formation, post-operative ileus occurrence, surgical site infection, re-operation and re-admission rates, length of hospital stay, and mortality rate.
- *Study design:* systematic review and meta-analysis for comparative studies. The following were excluded from our analysis: case series, case reports, letters to the editor, and non-comparative single-arm studies. Data presented as a conference abstract and available online were considered.

### 2.1 Search strategy

A comprehensive literature search was conducted using the following online electronic databases and clinical trial registers: PubMed, MEDLINE, ScienceDirect, Embase, Scopus, clinical trials.gov, and Cochrane Central Register of Controlled Trials [CENTRAL] up to and including October 1, 2022. No language restrictions or filters were applied. Furthermore, a manual search of reference lists and bibliographies in previous reviews was performed to identify additional studies.

We used a combination of the following search terms: ‘inflammatory bowel disease’, ‘IBD’, ‘Crohn’s disease’, ‘CD’, ‘ulcerative colitis’, ‘UC’, ‘ileocecal resection’, ‘ileo-colic resection’, ‘subtotal colectomy’, ‘total colectomy’, ‘colectomy’, ‘proctectomy’, ‘ileal pouch anal anastomosis’, ‘IPAA’, ‘robotic’, ‘laparoscopic’, ‘laparoscopy’, ‘minimally invasive surgery’. Three authors independently performed the search of the aforementioned databases, and two authors reviewed the extracted articles independently.

### 2.2 Eligibility and study selection criteria

Articles included in this analysis were based on the PICO[S] framework outlined above. Any published randomized controlled trials [RCTs] or comparative cohort studies [prospective/retrospective] meeting the eligibility criteria were considered eligible for inclusion. We excluded any study comparing outcomes in patients diagnosed with colorectal malignancies. Moreover, duplicate studies were excluded and the titles and abstracts of selected studies were evaluated for relevance independently by two authors. These records were then classified as included, excluded, or requiring further evaluation. The full-text of those articles [where available] matching our inclusion criteria were retrieved. Disagreements in the selection of studies were resolved by discussion and agreement between the reviewers. If the discrepancies remained unresolved, the authorship team were consulted to reach a consensus.

### 2.3 Data extraction and collection

An electronic spreadsheet [as per the Cochrane recommendations] for data extraction was created in Microsoft Excel. Following pilot-testing with randomly selected articles the spreadsheet was modified and adjusted accordingly to create a final version. Two reviewers independently extracted information from each of the studies including:

- Study-related data [author details, year of publication, geographical location of the corresponding author, publishing journal, study design, number of patients in each arm, and inclusion and exclusion criteria].
- Baseline demographic and clinical information of the study population.
- Primary and secondary outcome data.

Any disagreements during this process were resolved through discussion between the reviewers. If these remained the authorship team were consulted for resolution.

### 2.4 Assessment of bias

Our included studies by design were all observational and consequently the Newcastle–Ottawa Scale [NOS] was used to assess risk of bias.^19^ This quality assessment tool used for non-randomized studies in the interpretation of meta-analytical results is based on a ‘star system’. Studies are judged on three broad perspectives and include: the selection of the study groups; the comparability of these groups; and the ascertainment of either the exposure [case-control studies] or outcome [cohort studies] of interest.

We considered studies to be low-risk if the total NOS score was 9, medium-risk if the score was 7/8, and high-risk of bias if the score was <6. Any disagreement at this stage between the reviewers was resolved through consultation and involvement of a third author.

### 2.5 Statistical analysis

All statistical analysis in this review was performed using Review Manager [RevMan] 5.3 [Nordic Cochrane Centre, Cochrane Collaboration]. In instances where dichotomous outcomes were reported, the odds ratio [OR] was analysed using the Mantel–Haenszel method. Where 25% or more of our included studies reported zero events in both arms [comparison groups], the risk difference [RD] was calculated instead of the OR. The mean difference [MD] with 95% confidence intervals [CI] was used as the statistical measure for continuous outcomes. If mean values were not available for continuous outcomes, then the median and interquartile range [IQR] were extracted. Using the equation described by Hozo *et al*.^20^ these data were subsequently converted to estimate the mean and standard deviation [SD]. A random-effects model was used in all analyses.

A *p*-value of <0.05 or a 95% CI not including 0 [MD] or 1 [OR] were considered statistically significant. The *I*^2^ statistic using the Cochran Q test [χ^2^] was used to quantify between-study heterogeneity. High values of χ^2^ and *I*^2^ signify increasing levels of heterogeneity, with an *I*^2^ value of 0–40% representing low [might not be important], 30–60% moderate, 50–90% substantial, and 75–100% considerable heterogeneity.

To check possible causes of heterogeneity and evaluate the robustness of the results, subgroup analysis was performed for the individual operations performed [ileocaecal resection, total/sub-total colectomy, proctectomy with IPAA]. Moreover, sensitivity analysis was performed by calculating the risk ratio [RR] or RD for dichotomous variables and ‘leave one out’ analysis conducted to assess the effect of each study on the overall effect size and heterogeneity by repeating the analysis after excluding one study at a time.

## 3. Results

A total of 991 articles were identified after the systematic search of the above-mentioned electronic resources. Six were duplicates and, following their removal, 985 unique articles remained. A further 737 studies were excluded following review of their titles/abstracts. These included case reports, case series, letters, and articles not directly relevant to the topic.

Following this initial screening, 248 full-text articles were retrieved and reviewed. This process led to the exclusion of 237 studies as they compared interventions not relevant to the present analysis and those deemed ineligible for inclusion as they consisted of cohorts with malignant disease.

Eleven relevant articles^21–31^ were subsequently identified, of which two were conference abstracts^23,24^ and included in our final analysis. These were all non-randomized studies predominantly from the USA [*n* = 7] and published between 2012 and 2022.

A total of 5566 patients were divided between those undergoing robotic [*n* = 365] and conventional laparoscopic [*n* = 5201] surgery. In six studies an IPAA^21–26^ was created, ileo-colic resection was performed in three^27–29^ and sub-total colectomy in two.^30,31^

**Figure 1.**
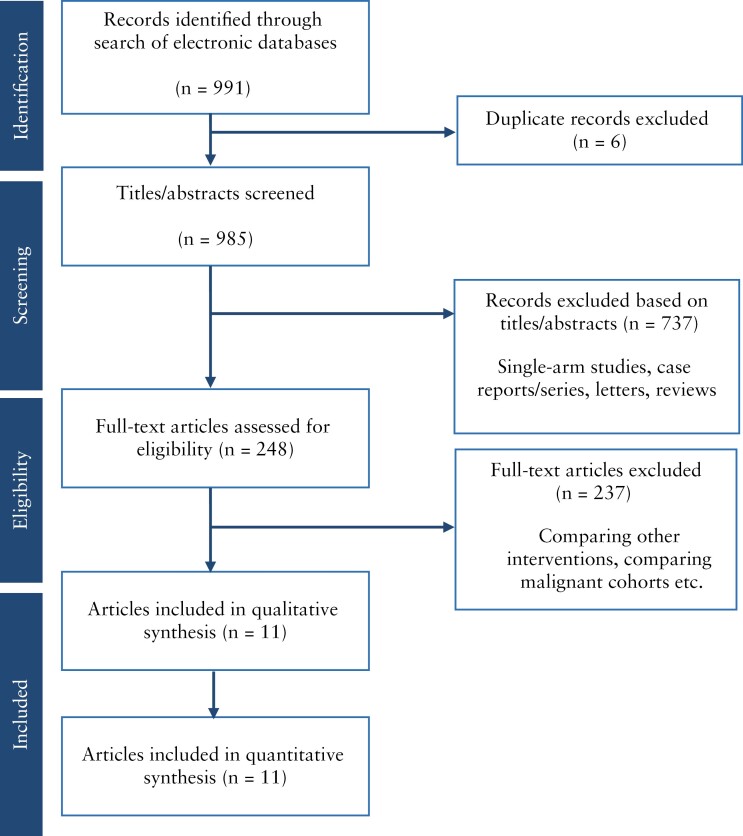
PRISMA flow chart.

The PRISMA flow chart is shown in Figure 1. Table 1 shows the baseline characteristics of the included studies. Table 2 shows the risk of bias assessed using the NOS.

**Table 1.** Baseline characteristics of the included studies

Study	Country	Study type	Number of patients	Inclusion and exclusion criteria	Operation
Miller 2012^21^	USA	Case-matched	Total: 34 Ro: 17 Lap: 17	Inclusion criteria: patients underwent surgery for UC, indeterminate colitis, or CD	Ileal J pouch-anal anastomosis for IBD
Rencuzogullari 2016^22^	USA	Prospective cohort	Total: 42 Ro: 21 Lap: 21	NR	Ileal J pouch-anal anastomosis for IBD
Marino 2018^23^	Italy	Case-matched	Total: 32 Ro: 16 Lap: 16	NR	Ileal J pouch-anal anastomosis for IBD
Elias 2019^24^	USA	Prospective cohort	Total: 116 Ro: 44 Lap: 72	NR	Ileal J pouch-anal anastomosis for IBD
Birrer 2022^25^	Switzerland	Retrospective cohort	Total: 27 Ro: 17 Lap: 10	Inclusion criteria: patients with a diagnosis of UC undergoing a three-stage restorative proctocolectomy. Exclusion criteria: patients with synchronous colorectal cancers and patients <18 years of age	Ileal J pouch-anal anastomosis for IBD
Gebhardt 2022^26^	Germany	Retrospective cohort	Total: 67 Ro: 29 Lap: 38	Inclusion criteria: age ≥18 years; medically refractory UC; elective surgery. Exclusion criteria: age ≤18 years; proven carcinoma or dysplasia; CD, indeterminate colitis, or familial adenomatous polyposis	Ileal J pouch-anal anastomosis for IBD
Aydinli 2020^27^	USA	Retrospective cohort	Total: 47 Ro: 33 Lap: 14	Inclusion criteria: patients who underwent ileocolic resection for CD with a purely robotic [R] or laparoscopic [L] approach	Ileocaecal resection for CD
Hota 2020^28^	USA	Retrospective cohort	Total: 3342 Ro: 121 Lap: 3221	Exclusion criteria: patients with ascites, disseminated cancer, ventilator dependence, sepsis, ASA class 5, and age ≥90 years	Ileocaecal resection for CD
Zambonin 2020^29^	Italy	Retrospective cohort	Total: 73 Ro: 10 Lap: 63	Inclusion criteria: patients with CD who had failure of medical treatment or the onset of complications	Ileocaecal resection for CD
Anderson 2019^30^	USA	Retrospective cohort	Total: 19 Ro: 6 Lap: 13	Exclusion criteria: patients younger than 18 years of age and any diagnosis other than UC	Sub-total colectomy for UC
Hota 2020^31^	USA	Retrospective cohort	Total: 1767 Ro: 51 Lap: 1716	Exclusion criteria: ascites, disseminated cancer, ventilator dependence, sepsis, American Society of Anesthesiologists [ASA] class 5, age ≥90 years	Sub-total colectomy for UC

Ro, robotic surgery; Lap, laparoscopic surgery; NR, not recorded; IBD, inflammatory bowel disease; CD, Crohn’s disease; UC, ulcerative colitis.

**Table 2.** Risk of bias assessment using the Newcastle–Ottawa Scale

Study	Representativeness of the exposed cohort	Selection of the non-exposed cohort	Ascertainment of exposure	Demonstration that outcome of interest was not present at start of the study	Comparability of cohorts based on the design or analysis controlled for confounders	Assessment of outcome	Was follow-up long enough for outcomes to occur?	Adequacy of follow-up of cohorts	Total score
Miller 2012^21^	*	*	*	*	*	*	*	*	8
Rencuzogullari 2016^22^	*	*	*	*	*	*		*	7
Marino 2018^23^	NA	NA	NA	NA	NA	NA	NA	NA	NA
Elias 2019^24^	NA	NA	NA	NA	NA	NA	NA	NA	NA
Birrer 2022^25^	*	*	*	*		*	*	*	7
Gebhardt 2022^26^	*	*	*	*	*	*	*	*	8
Aydinli 2020^27^	*	*	*	*		*	*		6
Hota [CD] 2020^28^	*	*	*	*		*	*	*	7
Zambonin 2020^29^	*	*	*	*		*	*	*	7
Anderson 2019^30^	*	*	*	*	*	*	*	*	8
Hota [UC] 2020^31^	*	*	*	*		*	*	*	7

CD, Crohn’s disease; UC, ulcerative colitis; NA, not available.

Three studies, scoring 8, were graded as low risk^21,26,30^ as assessed using the NOS. In two studies,^23,24^ as the abstract only was available [without access to the full article], risk of bias assessment was not possible and consequently not performed. In the remaining six articles,^22,25,27–29,31^ scores ranged between 6 and 7 [intermediate risk].

Comparability of cohorts based on the design or analysis controlled for confounders was only assessed in four studies^21,22,26,30^ using the NOS, as a matching technique in the selection of patients was employed.

### 3.1 Primary outcome

#### 3.1.1 Total complications

*All studies:* Ten studies^21–27,29–31^ with 2177 patients reported on total post-operative complications. The robotic surgery group [30.3%] showed a statistically lower rate of overall complications compared with the laparoscopy group [43.1%] (OR: 0.48, 95% CI [0.24, 0.93], *p* = 0.03). Cochran’s Q test revealed a moderate level of heterogeneity between the included studies [*I*^2^ = 52%, *p* = 0.03].

*Subgroup analysis:* This difference between our groups was not seen on subgroup analysis of the separate procedures: proctectomy and IPAA^21–26^ 31.9% [robotic surgery] vs 53.2% [laparoscopy group] (OR: 0.40, 95% CI [0.15, 1.11], *p* = 0.08); ileo-colic resection^27,29^ 9.3% [robotic surgery] vs 14.4% [laparoscopy group] (OR: 0.45, 95% CI [0.11, 1.87], *p* = 0.27); and sub-total colectomy^30,31^ 42.1% [robotic surgery] vs 44% [laparoscopy group] (OR: 0.79, 95% CI [0.25, 2.49], *p* = 0.69).

### 3.2 Secondary outcomes

#### 3.2.1 Total operative time [minutes]

*All studies:* Nine studies^21,22,24–28,30,31^ reported data on total operative time, and this was significantly shorter in the laparoscopic group compared with robotic surgery (MD: 40.61, 95% CI [24.23, 56.99], *p* = 0.00001). Cochran’s Q test revealed a substantial level of between-study heterogeneity [*I*^2^ = 73%, *p* = 0.0002].

*Subgroup analysis:* The difference in operative time between the two groups remained even after subgroup analysis separated out the individual procedures and was as follows: proctectomy and IPAA^21,22,24–26^ (MD: 38.07, 95% CI [7.14, 68.99], *p* = 0.02); ileocolic resection^27,28^ (MD: 46.21, 95% CI [12.52, 79.90], *p* = 0.007); and sub-total colectomy^30,31^ (MD: 51.46, 95% CI [37.43, 65.50], *p* < 0.00001). Cochran’s Q test revealed considerable heterogeneity between the included studies for proctectomy and IPAA [*I*^2^ = 78%, *p* = 0.001] and ileocolic resection [*I*^2^ = 73%, *p* = 0.05] and low-level heterogeneity for sub-total colectomy [*I*^2^ = 0%, *p* = 0.41].

#### 3.2.2 Conversion to open surgery

*All studies:* Eight studies^21–23,25–27,29,30^ with a total of 354 patients reported on conversion rates. Pooled analysis showed a non-statistically significant trend to lower conversion rate in the robotic surgery group [4%] compared with conventional laparoscopy [11.2%] (OR: 0.46, 95% CI [0.16, 1.32], *p* = 0.15). Cochran’s Q test revealed a low level of between-study heterogeneity [*I*^2^ = 0%, *p* = 0.79].

*Subgroup analysis:* Performing subgroup analysis on conversion rates in proctectomy and IPAA^21–23,25,26^ and ileocolic resection^27,29^ also showed no statistically significant difference between the two groups. The statistical measures for proctectomy and IPAA conversion rates were 5% [robotic surgery] compared with 8.8% [laparoscopic surgery] (OR: 0.54, 95% CI [0.16, 1.82], *p* = 0.32] and for ileocolic resection 2.3% [robotic surgery] compared with 15.5% [laparoscopic group] (OR: 0.30, 95% CI [0.04, 2.31], *p* = 0.25), respectively.

#### 3.2.3 Anastomotic leak rate

*All studies:* Six studies^21,22,27–29,31^ with 5345 patients reported on anastomotic leaks. This outcome was comparable between the two groups with 2.3% [robotic surgery] and 2.9% [laparoscopic group] (OR: 0.92, 95% CI [0.40, 2.10], *p* = 0.84). A low level of heterogeneity was found between the included studies [*I*^2^ = 0%, *p* = 0.89].

*Subgroup analysis:* Comparable anastomotic leak rates were also noted between the groups following subgroup analysis in patients undergoing proctectomy and IPAA^21,22^ (OR: 0.95, 95% CI [0.13, 6.91], *p* = 0.96) and ileocolic resection^27–29^ (OR: 0.99, 95% CI [0.38, 2.60], *p* = 0.99), respectively.

#### 3.2.4 Abdominal abscess/collection formation

*All studies:* Four studies^21,22,25,30^ [*n* = 122 patients] reported on post-operative intra-abdominal abscess occurrence. The robotic surgery group showed a lower rate of intra-abdominal abscess formation [1.6% compared with 8.2%], but this difference failed to reach statistical significance (OR: 0.34, 95% CI [0.06, 1.83], *p* = 0.21). Cochran’s Q test revealed a low level of between-study heterogeneity [*I*^2^ = 0%, *p* = 0.90].

*Subgroup analysis:* Subgroup analysis for proctectomy and IPAA^21,22,25^ alone also showed no significant difference when the procedure is performed through robotic platforms compared with conventional laparoscopy with rates of 1.8% versus 6.3%, respectively (OR: 0.33, 95% CI [0.05, 2.43], *p* = 0.28).

#### 3.2.5 Paralytic ileus post-procedure

*All studies:* Seven^21,22,25,27,28,30,31^ of our included studies with a total of 5305 patients reported post-operative paralytic ileus as an outcome. These rates were the same in both groups [22.9%] (OR: 1.35, 95% CI [0.98, 1.86], *p* = 0.06). Cochran’s Q test revealed a low level of heterogeneity between the studies [*I*^2^ = 0%, *p* = 0.80].

*Subgroup analysis:* Subgroup analysis showed that the rate of paralytic ileus was comparable between the groups when analysed for proctectomy and IPAA^21,22,25^ (OR: 0.96, 95% CI [0.28, 3.25], *p* = 0.95); ileo-colic resection^27,28^ (OR: 1.25, 95% CI [0.86, 1.83], *p* = 0.24); and sub-total colectomies^30,31^ (OR: 1.99, 95% CI [0.98, 4.05], *p* = 0.06).

#### 3.2.6 Surgical site infection

*All studies:* Surgical site infections [SSIs] were reported as an outcome in seven studies.^21,22,27–31^ This was low in both groups, 0.8% [robotic surgery] compared with 0.9% [laparoscopic group], with no statistical significance between them (OR: 0.93, 95% CI [0.58, 1.51], *p* = 0.78). A low level of heterogeneity was reported in the included studies [*I*^2^ = 0%, *p* = 0.71].

*Subgroup analysis:* The difference between our groups remained comparable following subgroup analysis evaluating proctectomy and IPAA,^21,22^ ileo-colic resection,^27–29^ and sub-total colectomy^30,31^ (OR: 0.78 95% CI [0.19, 3.25], *p* = 0.74; OR: 0.77, 95% CI [0.40, 1.49], *p* = 0.43; and OR: 1.33, 95% CI [0.59, 3.00], *p* = 0.49, respectively).

#### 3.2.7 Re-operation rate

*All studies:* Re-operation rate was reported as an outcome in nine studies^22,24–31^ [*n* = 5540 patients]. No significant difference was detected between the two groups with 5.1% in the robotic surgery group compared with 3.9% in the laparoscopic group (OR: 1.42, 95% CI [0.77, 2.60], *p* = 0.26).

A low level of heterogeneity was reported between the included studies [*I*^2^ = 0%, *p* = 0.89].

*Subgroup analysis:* No statistically significant difference was noted on subgroup analysis either of this outcome for proctectomy and IPAA^22,24–26^ (OR: 2.69, 95% CI [0.76, 9.49], *p* = 0.12), ileo-colic resection^27–29^ (OR: 1.07, 95% CI [0.40, 2.81], *p* = 0.90), and sub-total colectomy^30,31^ (OR: 1.28, 95% CI [0.48, 3.44], *p* = 0.63).

#### 3.2.8 Re-admission to hospital

*All studies:* Four studies^22,25,27,30^ with a total of 135 patients reported on re-admission rates. This outcome was 12.9% in the robotic surgery group compared with 20.7% in the laparoscopic group. There was no statistically significant difference between the groups (OR: 0.70, 95% CI [0.26, 1.89], *p* = 0.48). Cochran’s Q test revealed a low level of heterogeneity between our included studies [*I*^2^ = 0%, *p* = 0.75].

*Subgroup analysis:* On subgroup analysis for patients undergoing proctectomy and IPAA,^22,25^ the results remained comparable with no significant difference between the robotic and laparoscopic approaches (OR: 1.10, 95% CI [0.30, 3.96], *p* = 0.89).

#### 3.2.9 Length of stay

*All studies:* Length of hospital stay [LOS] was reported in nine studies^21,22,24–27,29–31^ and although favouring robotic surgery, was not significantly different between the groups (MD: −0.19, 95% CI [−0.81, 0.44], *p* = 0.56). A low level of heterogeneity was reported on Cochran’s Q test [*I*^2^ = 33%, *p* = 0.15].

*Subgroup analysis:* More detailed analysis showed comparable LOS between the two groups for proctectomy and IPAA^21,22,24–26^ and ileo-colic resections^27,29^ (MD: 0.22, 95% CI [−0.93, 1.36], *p* = 0.71; and MD: 0.09, 95% CI [−0.64, 0.82], *p* = 0.81, respectively). However, LOS was significantly shorter following robotic sub-total colectomy compared with conventional laparoscopy^30,31^ (MD: −1.62, 95% CI [−3.11, −0.13], *p* = 0.03).

#### 3.2.10 Mortality rate

*All studies:* Thirty-day mortality was reported as an outcome in six studies^21,22,24,25,27,30^ with a total of 285 patients. The mortality rate was 0% in the robotic and laparoscopic groups (RD: 0.00, 95% CI [−0.03, 0.03], *p* = 1.00). Cochran’s Q test revealed a low level of between-study heterogeneity [*I*^2^ = 0%, *p* = 1.00].

**Figure 2.**
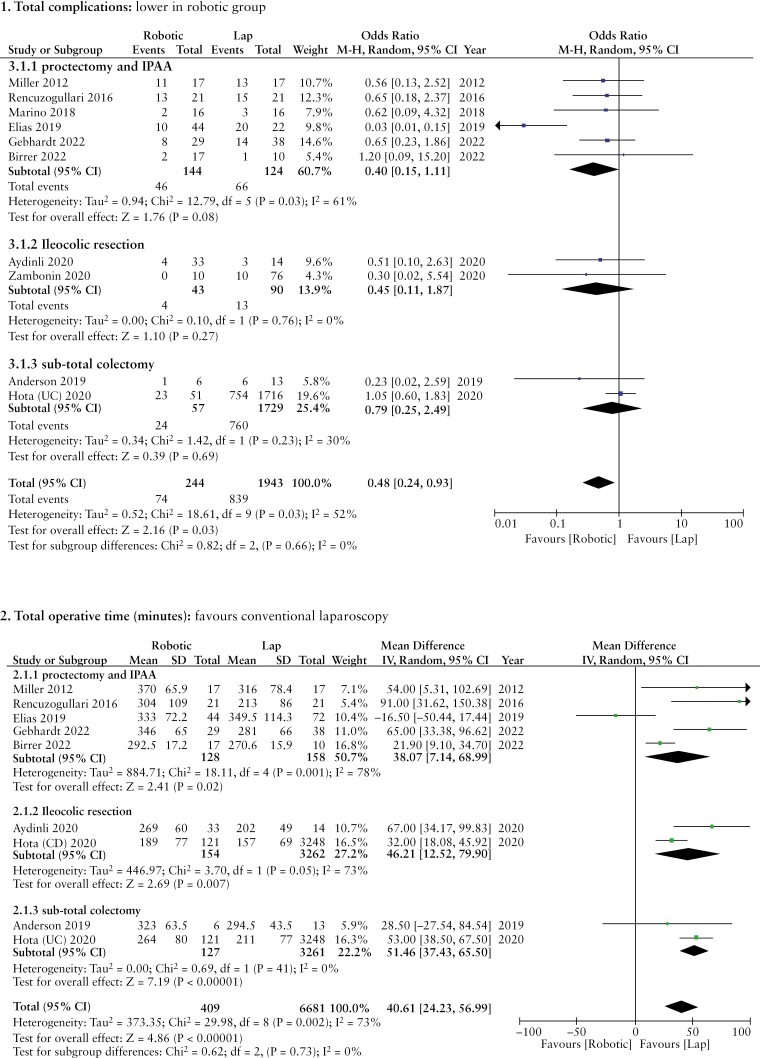

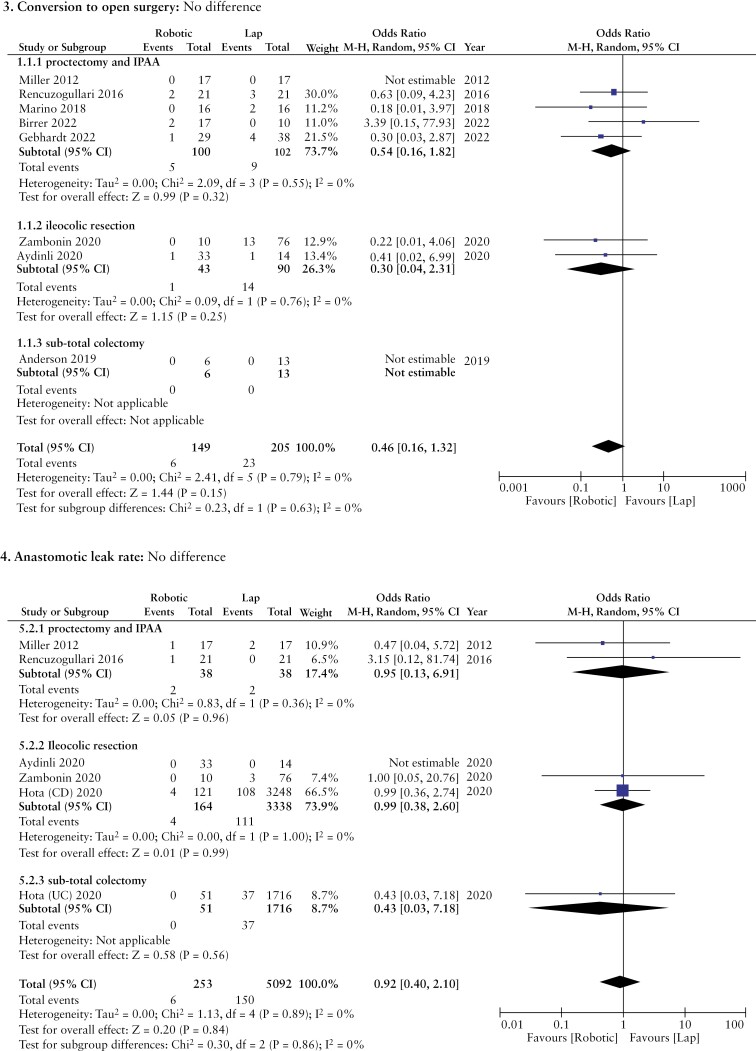

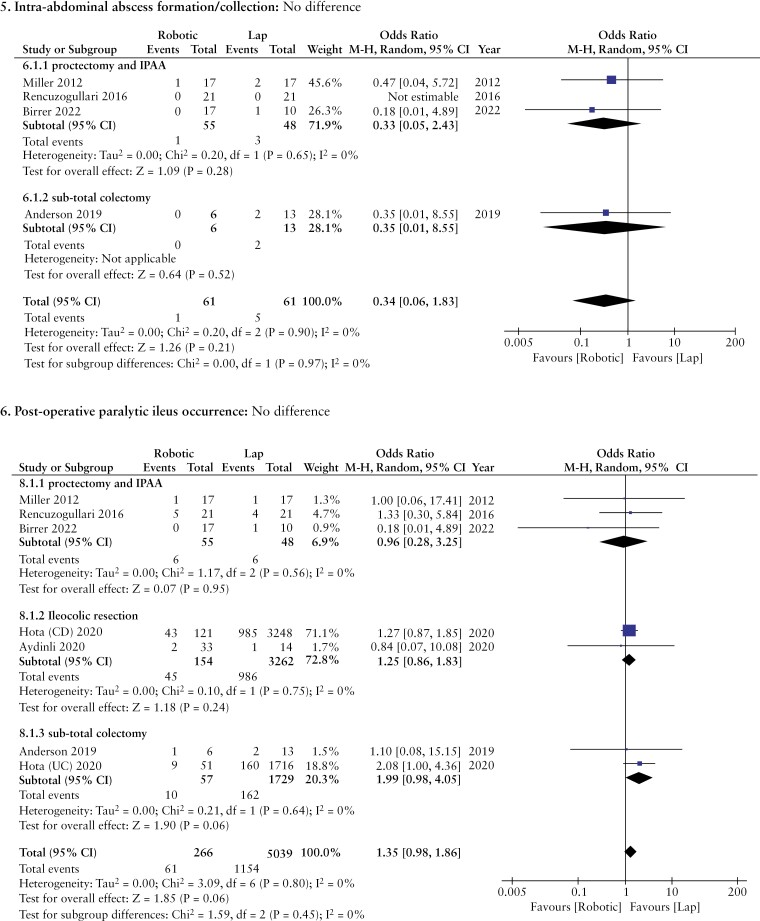

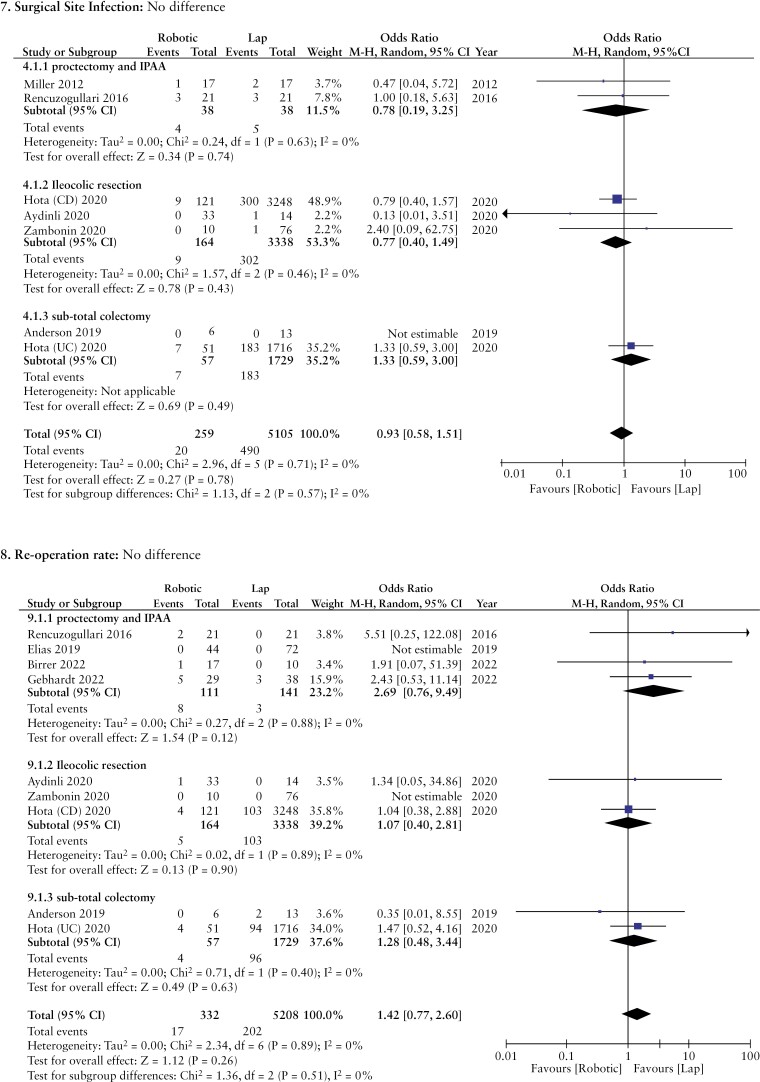

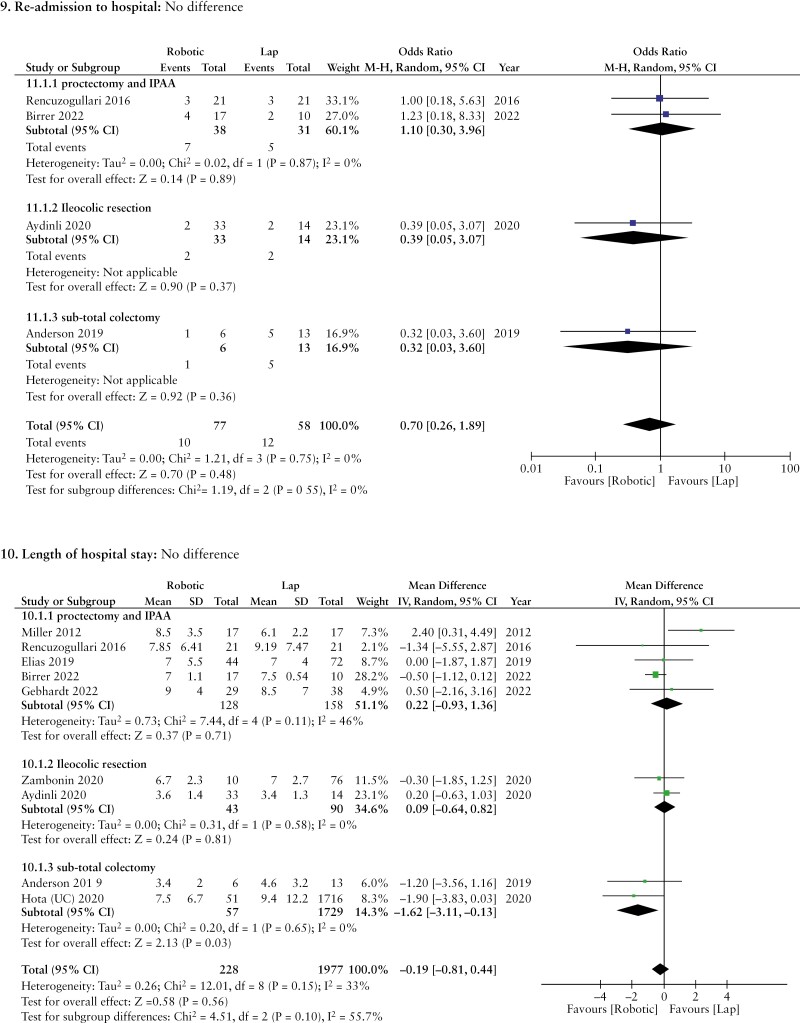

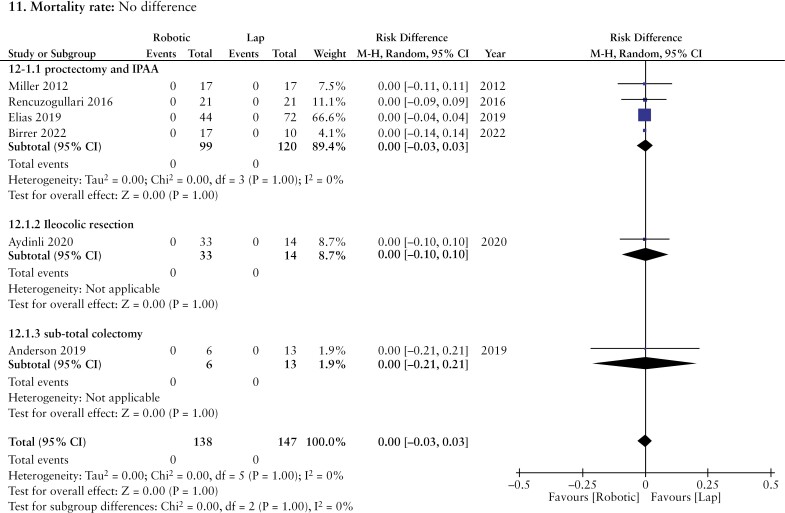
Forest plots of comparison of [1] total complications, [2] total operative time [minutes], [3] conversion to open surgery, [4] anastomotic leak rate, [5] intra-abdominal abscess/collection formation, [6] post-operative ileus occurrence, [7] surgical site infection, [8] re-operation rate, [9] re-admission to hospital, [10] length of hospital stay, and [11] mortality rate. Solid squares denote the mean difference, odds ratio, or risk difference. Horizontal lines represent the 95% confidence intervals [CIs], and the diamond denotes the pooled effect size. Lap, laparoscopic; M–H, Mantel–Haenszel test.

Figure 2 shows the associated forest plots.

## 4. Discussion

This systematic review and meta-analysis of 11 observational studies^21–31^ compared laparoscopic and robotic surgical techniques for IBD. The primary outcome was total complications and this study showed a significantly lower rate in patients undergoing robotic surgery as compared with standard laparoscopic techniques. Total procedure times tended to be longer in the robotic group compared with the conventional laparoscopy group. However, other secondary outcomes including conversion rates, intra-abdominal abscess formation, and anastomotic leak rates were comparable.

To our knowledge, this is the first meta-analysis of its kind looking at the breadth of IBD and associated surgical procedures. The results need to be interpreted with the knowledge that the available published data analysed in this study are of low quality. Robotic resections seem to be technically feasible with at least comparable outcomes during the early learning curve of using these novel surgical techniques.

This study focused solely on patients with an established diagnosis of IBD and included a wide range of surgical procedures such as ileo-colic resections, sub-total colectomies, and proctectomy with or without IPAA. Intra-abdominal abscess/collection formation, as a secondary outcome, was only reported in one study^27^ in the sub-total colectomy group and not reported as an outcome measure in patients undergoing ileo-colic resections. We were therefore unable to determine if there were any sub-group differences.

Heterogeneity in the reported outcomes and limited recording of quality of life [QoL] and functional bowel outcomes makes formal data synthesis and comparisons challenging. Sensitivity analyses were unable to reduce this. The lack of long-term follow-up data is another limiting factor.

The advantages offered by robotic platforms including high definition 3-D views, tremor reduction, and the availability of wristed instruments are all of use to surgeons operating in confined spaces including the pelvis.^32^ With its promise of more precise dissection in embryological planes and preservation of pelvic nerves, robotic surgery may help reduce tissue trauma further leading to quicker post-operative recovery and potentially fewer long-term complications compared with standard laparoscopy. However, more studies on long-term functional bowel outcomes and cost–benefit analyses are needed to fully assess the impact of robotic surgery.

As robotic surgery is an evolving discipline in the field of IBD surgery and given the ‘learning curve’ associated with the introduction of novel techniques, perhaps unsurprisingly total operative time at present is longer compared with other minimally invasive procedures. This can have a significant impact on the cost of a procedure. However, as would be expected with any new technique growing experience and familiarity should help to reduce operating time.^33^

A previous review comparing robotic versus laparoscopic restorative proctocolectomy in benign disease reported significantly longer operative time in robotic cases. However, estimated blood loss and duration of hospital stay was significantly less in some studies and a trend to fewer complications with robotic surgery was reported with little difference in functional and QoL outcomes.^16^

In robotic ileo-colic resection with intracorporeal anastomosis for CD, time to recovery of bowel function post-operatively was significantly shorter compared with laparoscopy. Consistent with our findings, mean operative time was significantly longer [51 min] in the robotic group but the authors attributed this to ureteric stent insertion in approximately one-third of these patients. A non-statistically significant trend to fewer conversions to open surgery was seen compared with the laparoscopic group. Other analysed outcomes including complication and re-operation rates were similar for the two approaches.^27^

In their study the authors suggested that due to technical challenges intracorporeal anastomosis is not widely performed in conventional laparoscopy.^27^ Easier suturing with wristed instruments offered by robotic platforms may help to overcome this limitation and facilitate construction of intracorporeal anastomosis.

Potential benefits derived from this technique as a consequence of ‘off-midline’ Pfannenstiel incision specimen extraction compared with midline extracorporeal anastomosis have been suggested to include better cosmesis, reduction in post-operative pain, reduction in the risk of incisional hernia occurrence, and faster recovery of bowel function.

The authors concluded that the robotic approach is safe and feasible in complex CD. Advantages are gained through increased dexterity offered by endo-wristed instruments, magnified high-resolution 3-D views, capacity for multi-quadrant surgery, and the use of energy devices especially in the presence of thickened, friable tissue.^27^

The Minimally Invasive Right Colectomy Anastomosis [MIRCAST]^34^ study recently published a comparison between laparoscopic, robotic, intracorporeal, and extracorporeal anastomoses during right hemicolectomies. The results showed positive trends towards robotic intracorporeal anastomosis and these can be extrapolated for IBD surgery.

Meta-analysis of observational studies is not without limitations that need to be considered. One of the main limitations is the potential to introduce bias. Observational studies are not randomized, and therefore there may be confounding variables that affect our results. Additionally, the quality of the studies included in the meta-analysis varies, impacting results and data interpretation.

Moreover, high levels of heterogeneity existed between the included studies for some outcome measures and sensitivity analysis was unable to reduce this. The lack of long-term follow-up data in the included studies makes it difficult to draw robust conclusions between the techniques.

Additionally, important outcome measures such as return of gut function, disease recurrence, and survival data were not reported, thereby limiting the quality of this review. Functional outcomes and QoL parameters were inconsistently reported to allow meaningful analysis. Finally, the available evidence from the current observational studies is subject to confounding by indication.

## 5. Conclusion

Outcomes in the surgical management of IBD are comparable between laparoscopic techniques and robotic-assisted surgery, demonstrating the safety and feasibility of robotic platforms. Fewer total complication rates are noted using the robotic approach. Larger studies investigating the use of robotic technology in CD and UC separately may be of benefit with a specific focus on important IBD-related metrics and outcomes.

## Data Availability

All data relevant to this study are included in the article. Additional data are available on request from the corresponding author.
